# Supplementary material for: A combined high-throughput and high-content platform for unified on-chip synthesis, characterization and biological screening
Source: Nat Commun. 2020 Oct 26;11:5391. doi: 10.1038/s41467-020-19040-0 (PMC7589500; doi:10.1038/s41467-020-19040-0)
Supplement: Supplementary file 1 — Supplementary Information [file 41467_2020_19040_MOESM1_ESM.pdf]

Supplementary Information for

A combined high-throughput and high-content platform for unified on-chip synthesis, characterization and biological screening

Benz et al.

Supplementary Figures

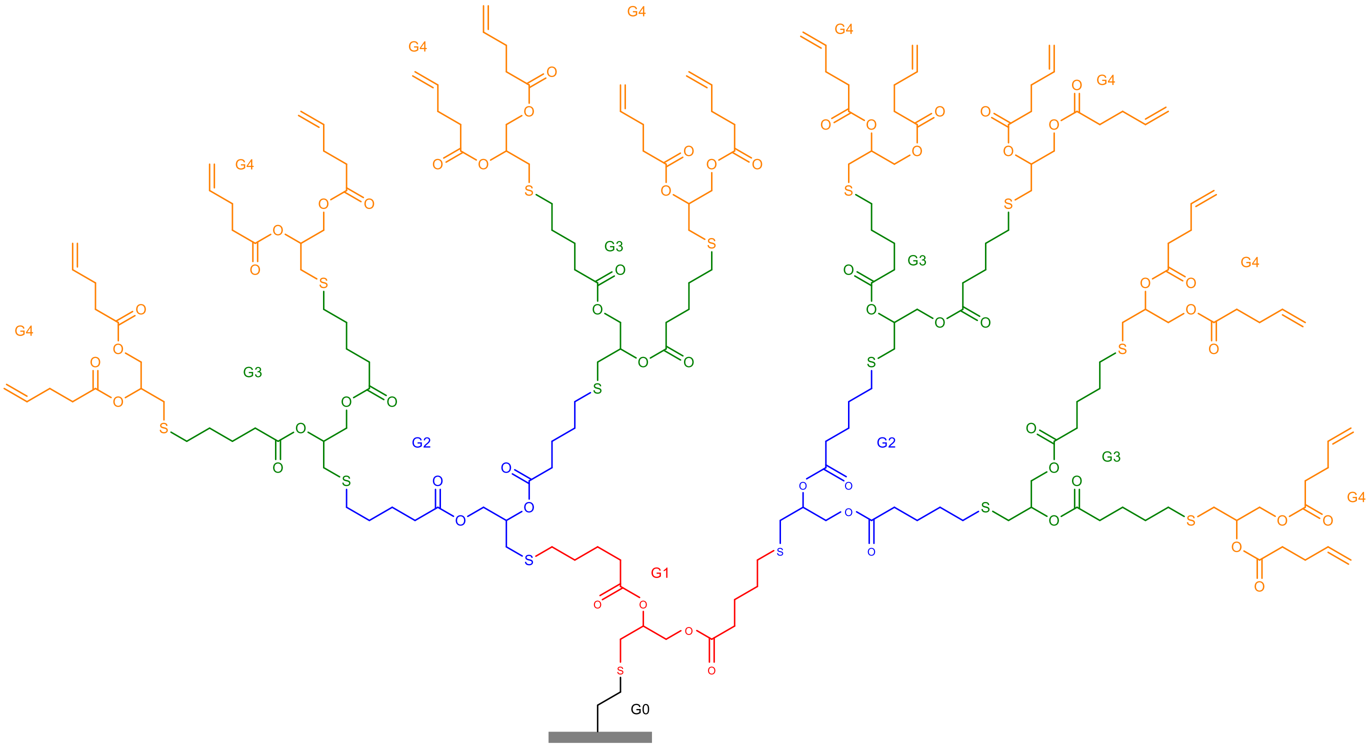

Supplementary Figure 1 | Chemical structure of a surface-grafted G4 dendrimer.

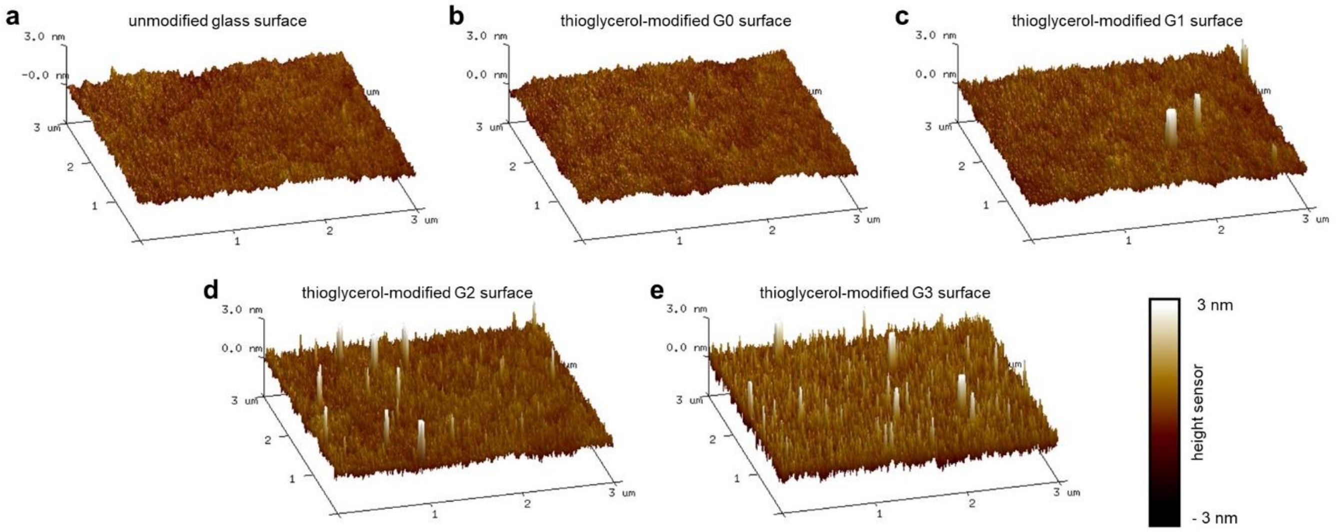

Supplementary Figure 2 | Atomic force microscopy (AFM) surface characterization. AFM image of an (**a**) unmodified, (**b**) thioglycerol-modified G0, (**c**) G1, (**d**) G2, and (**e**) G3 surface measured in non-contact tapping mode in air. Field-of-view: 3 x 3 nm^2.

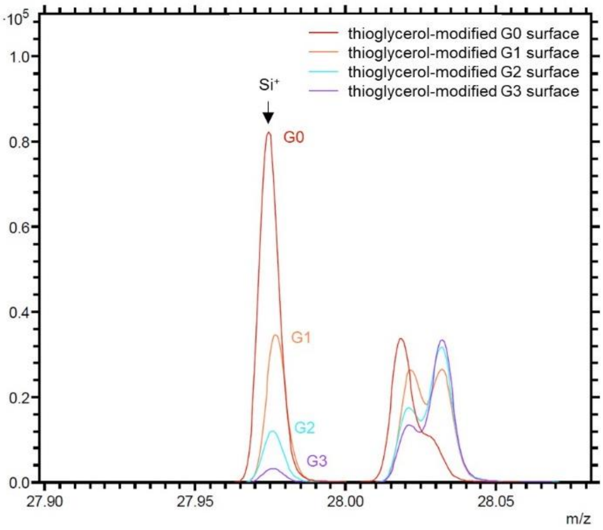

Supplementary Figure 3 | Time-of-flight secondary ion mass spectrometry (ToF-SIMS) surface characterization. ToF-SIMS results comparing the Si^+ signal intensity in positive polarity mode of thioglycerol-modified G0-3 surfaces. Source data are provided as a Source Data file.

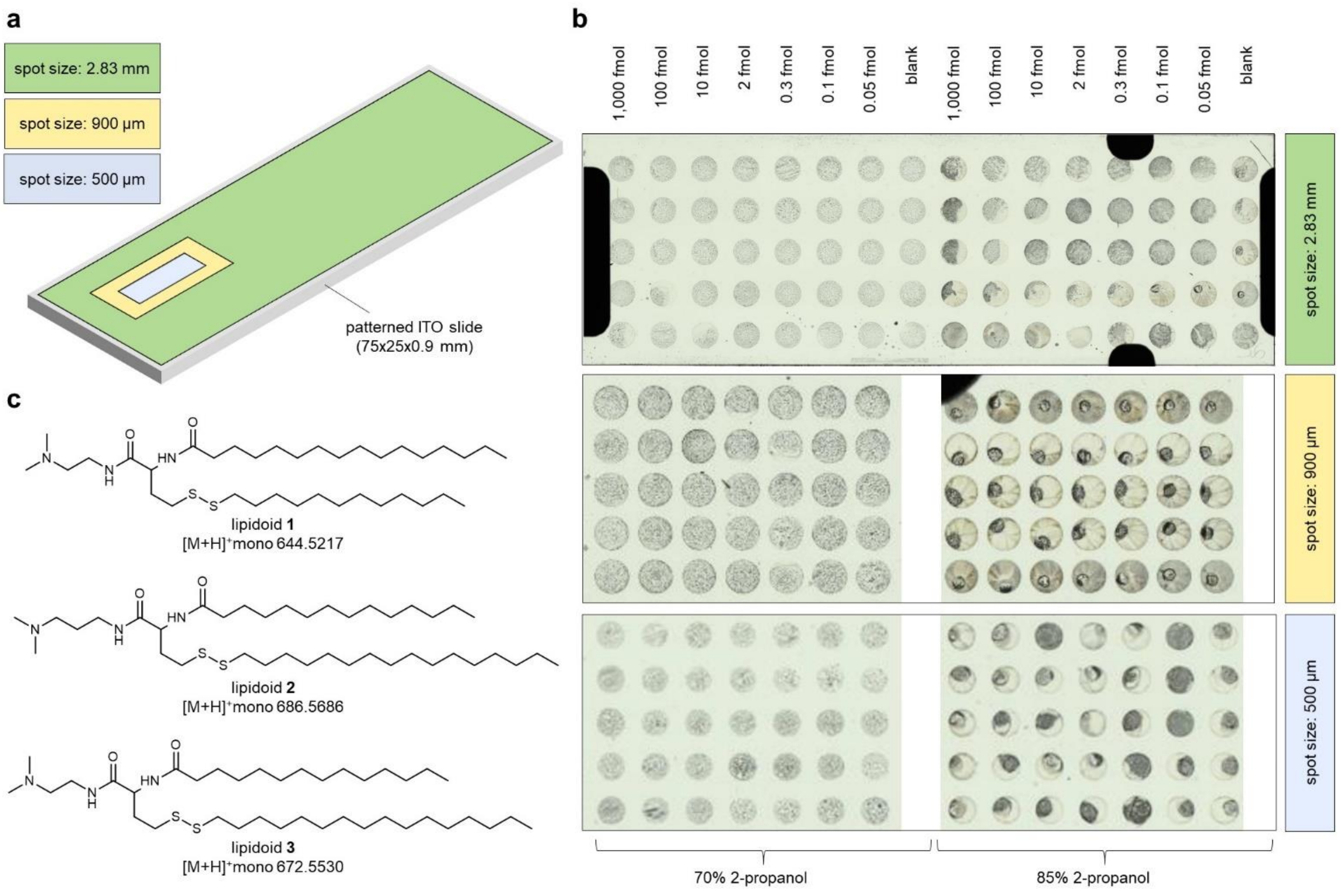

Supplementary Figure 4 | On-chip matrix-assisted laser desorption / ionization mass spectrometry (MALDI-TOF MS). (**a**) Schematically comparing the required surface area on a omniphilic-omniphobic patterned ITO slide for applying lipidoid **1** in seven different concentrations, each in five replications, and 2x five blank spots (CHCA matrix without sample) on 2.83 mm (spot width: 1.67 mm), 900 µm (spot width: 225 µm) and 500 µm (spot width: 250 µm) round spots. (**b**) Microscopy images of omniphilic-omniphobic patterned ITO slides with different spot sizes (2.83 mm, 900 µm and 500 µm round spots) containing dried lipidoid **1** in different amounts of substance per spot (1,000, 100, 10, 2, 0.3, 0.1 and 0.05 fmol). Matrix solution was applied using 70 and 85% 2-propanol. (**c**) Chemical structure of lipidoid **1**, **2** and **3**.

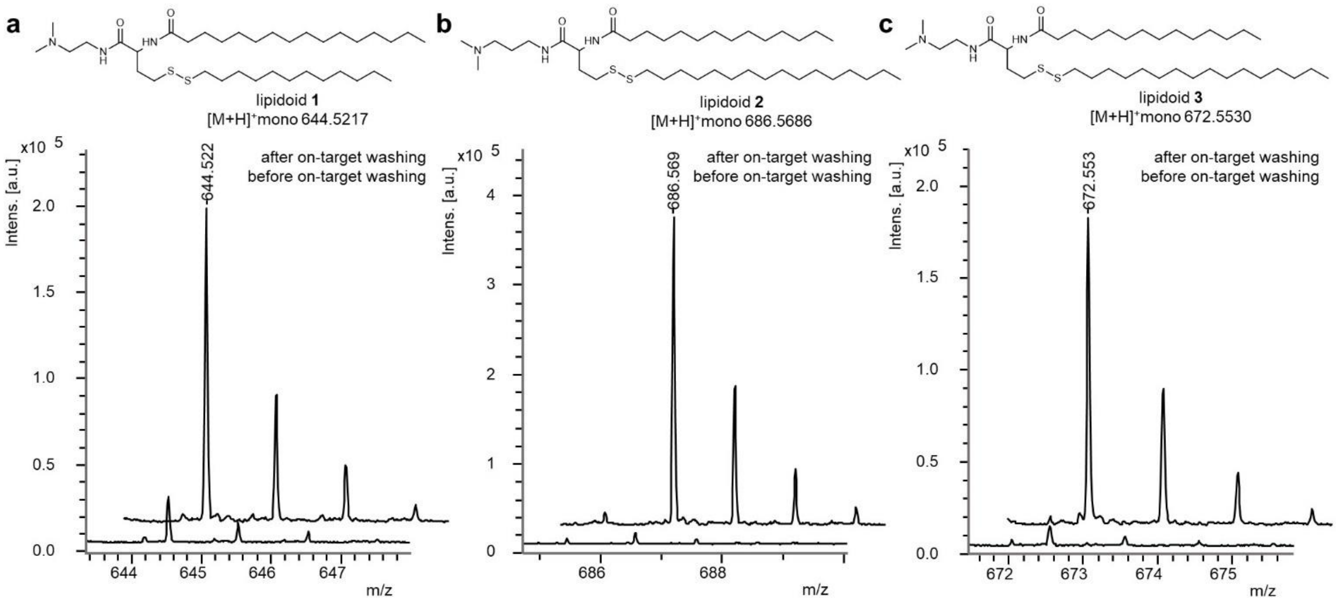

Supplementary Figure 5 | Effect of on-target washing on MALDI-TOF MS sensitivity. MS spectra of (**a**) lipidoid **1** ($[M+H]^+$mono: 644.5217), (**b**) lipidoid **2** ($[M+H]^+$mono: 686.5686) and (**c**) lipidoid **3** ($[M+H]^+$mono: 672.5530) before and after on-target washing. Source data are provided as a Source Data file.

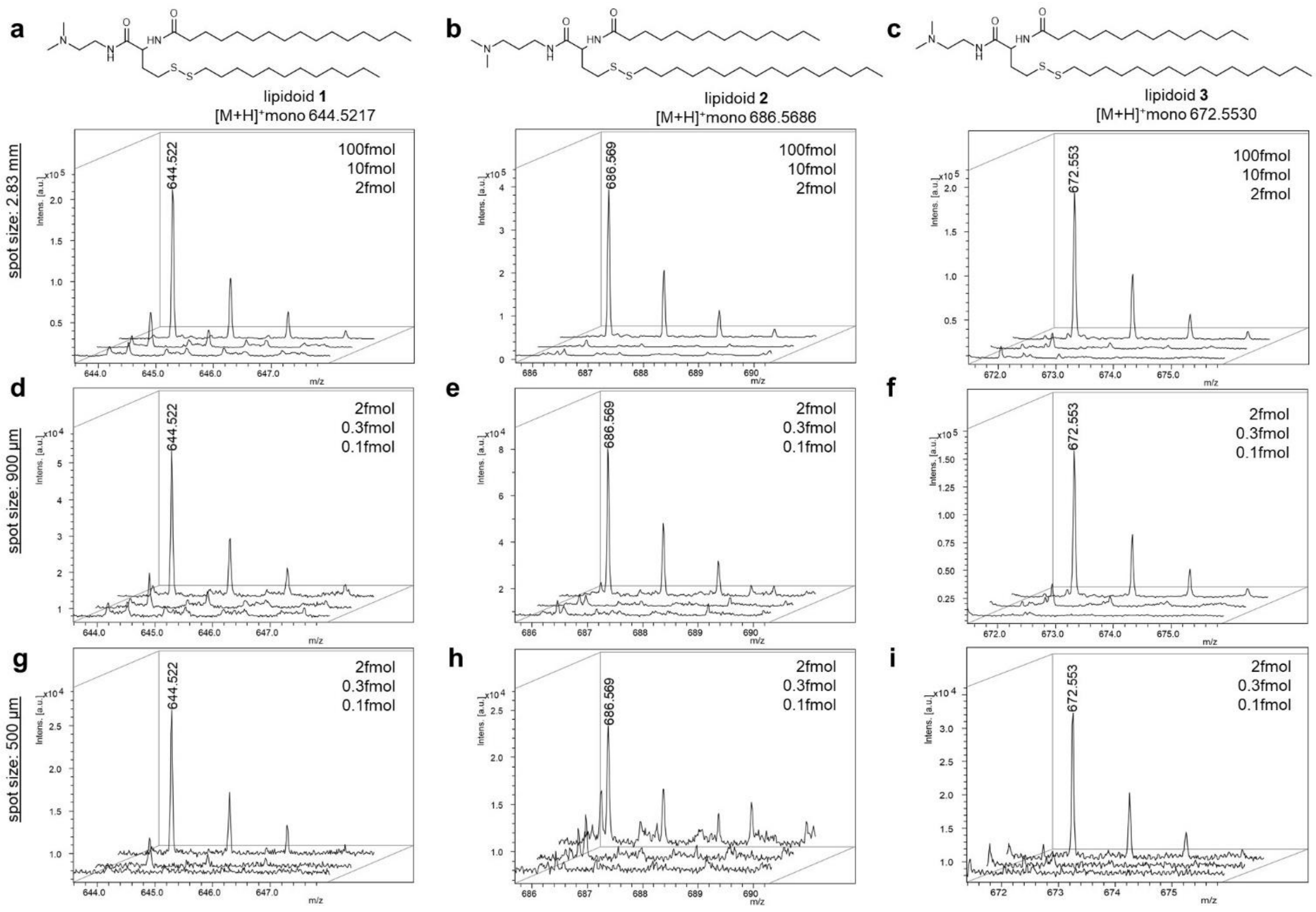

Supplementary Figure 6 | Sensitivity limitation of on-chip MALDI-TOF MS. MS spectra of lipidoid **1**, **2** and **3** of (**a-c**) 100, 10 and 2 fmol per 2.83 mm spot, (**d-f**) 2, 0.3 and 0.1 fmol per 900 µm spot, and (**g-i**) 2, 0.3 and 0.1 fmol per 500 µm spot. Source data are provided as a Source Data file.

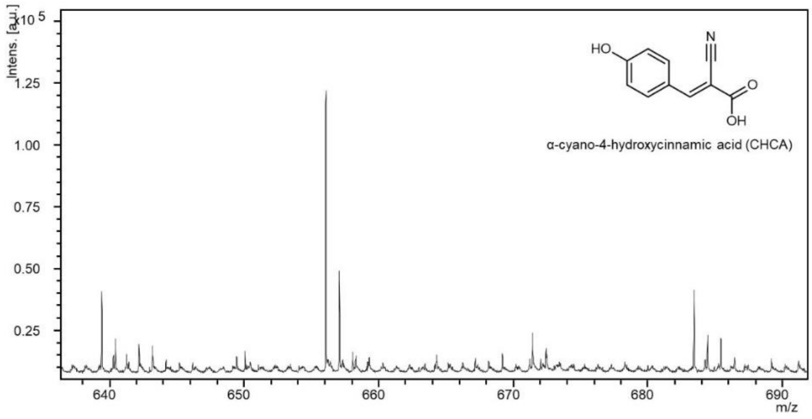

Supplementary Figure 7 | MALDI-TOF MS spectrum of α-cyano-4-hydroxycinnamic acid (CHCA) measured on-chip. Source data are provided as a Source Data file.

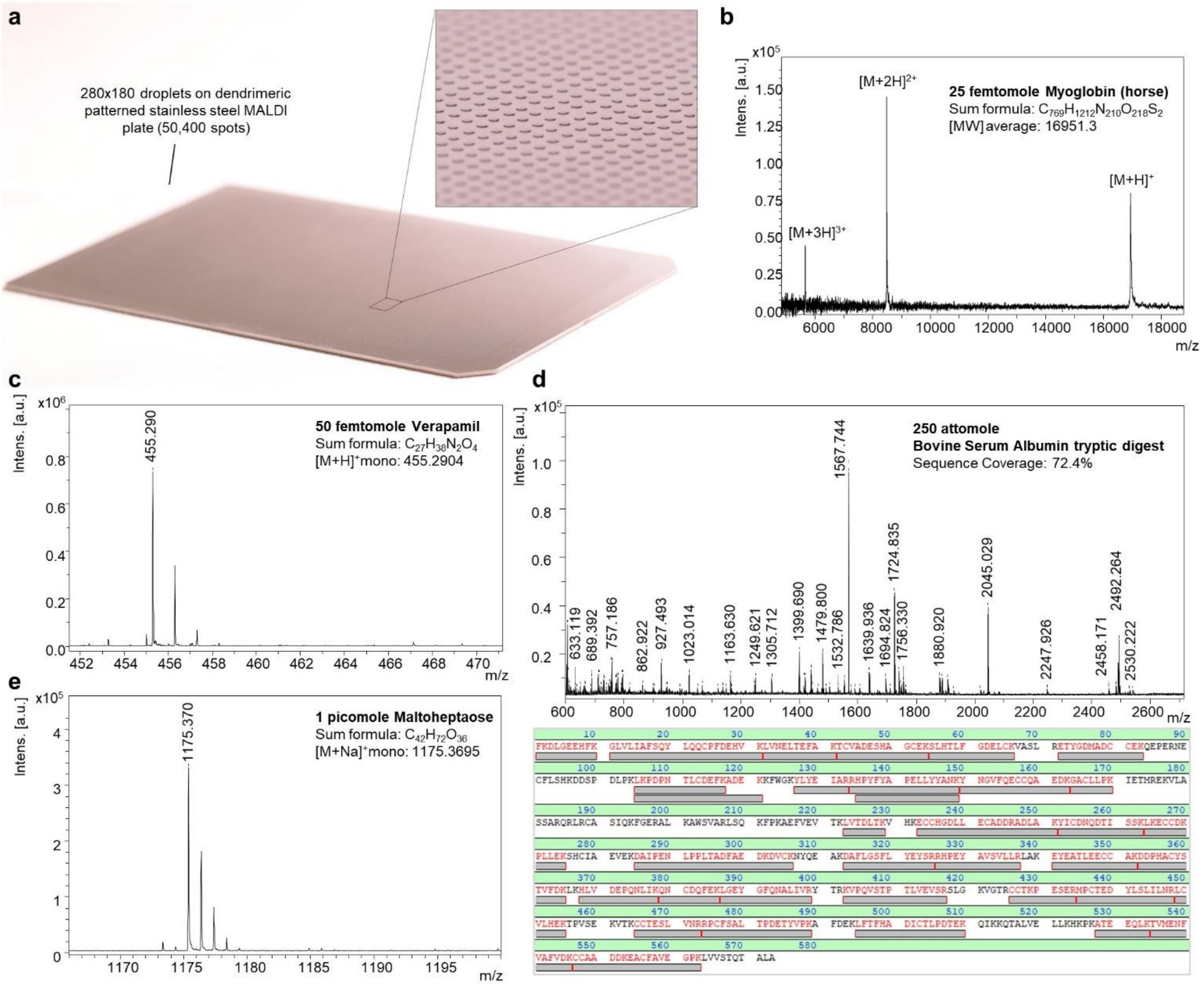

Supplementary Figure 8 | MALDI-TOF MS on an omniphilic-omniphobic patterned stainless steel plate. (**a**) Photograph of a dendrimer-modified and patterned stainless steel plate of microtiter plate size presenting 50,400 individual droplets. Spot size: 330x330 µm^2; spot distance: 60 µm; solvent: DMSO. Mass spectra measured on patterned stainless steel plate of (**b**) 25 femtomole myoglobin, (**c**) 50 femtomole Verapamil, (**d**) 250 attomole tryptic digested bovine albumin and (**e**) 1 picomole maltoheptaose.

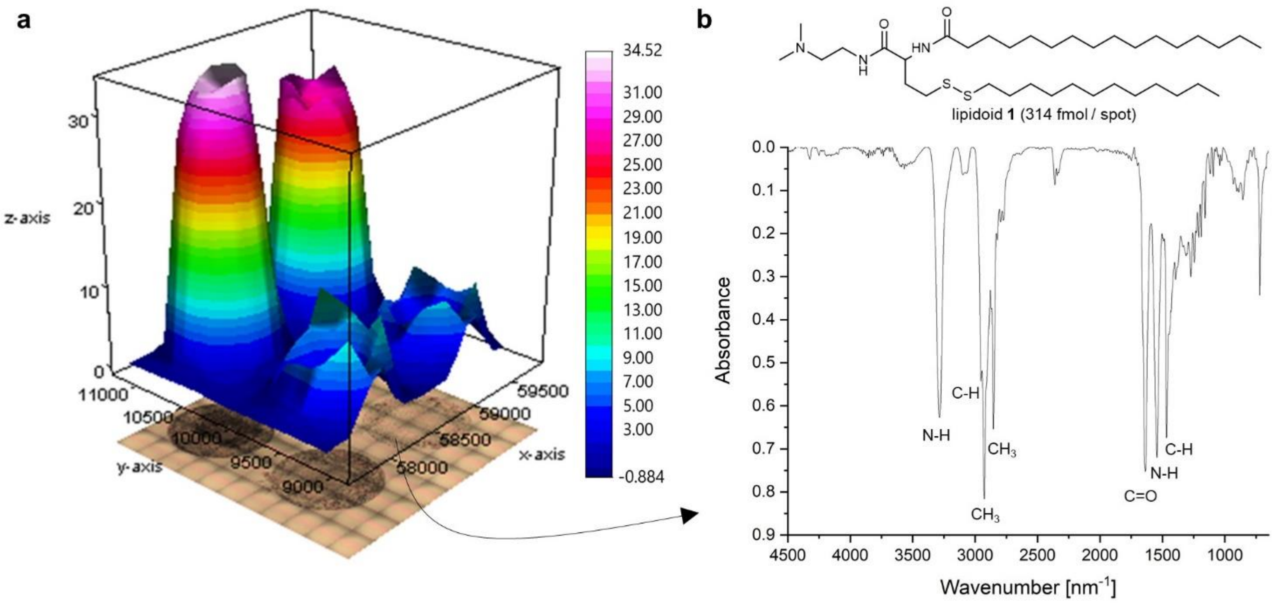

Supplementary Figure 9 | On-chip characterization by IR spectroscopy. (**a**) IR imaging of lipidoid **1** applied in different concentrations on an omniphilic-omniphobic patterned ITO surface. Color scale: Absorbance [a.u.]. (**b**) Corresponding on-chip measured IR spectrum of 314 fmol of lipidoid **1**. Source data are provided as a Source Data file.

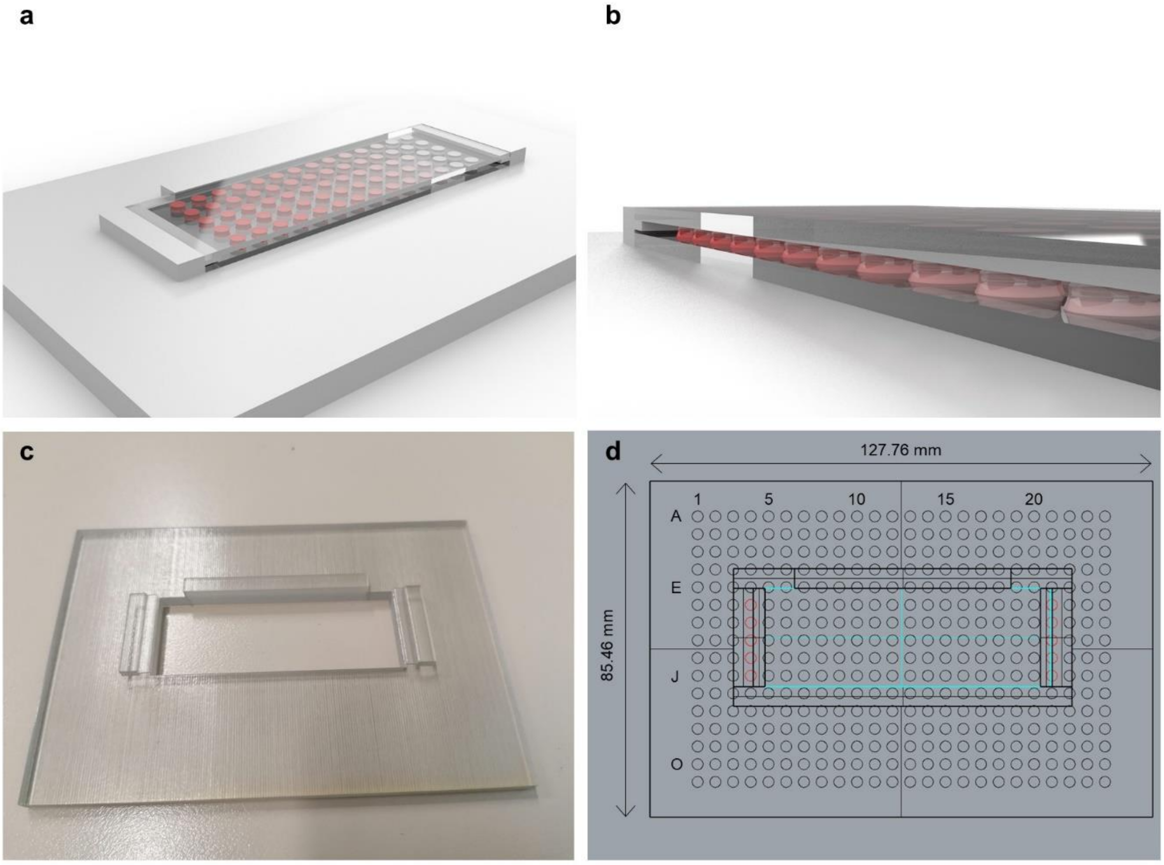

Supplementary Figure 10 | 3D-printed sandwiching adapter for UV-Vis measurements. (**a-b**) Concept art images of a droplet array trapped between two dendrimer-modified, omniphilic-omniphobic patterned slides. The slides were sandwiched using a 3D-printed adapter. (**c**) Photography of the 3D-printed sandwiching adapter. (**d**) Schematically showing the top view of the sandwiching adapter with overlaid 384-well microtiter plate patterns. Blue: omniphilic-omniphobic patterned glass slide.

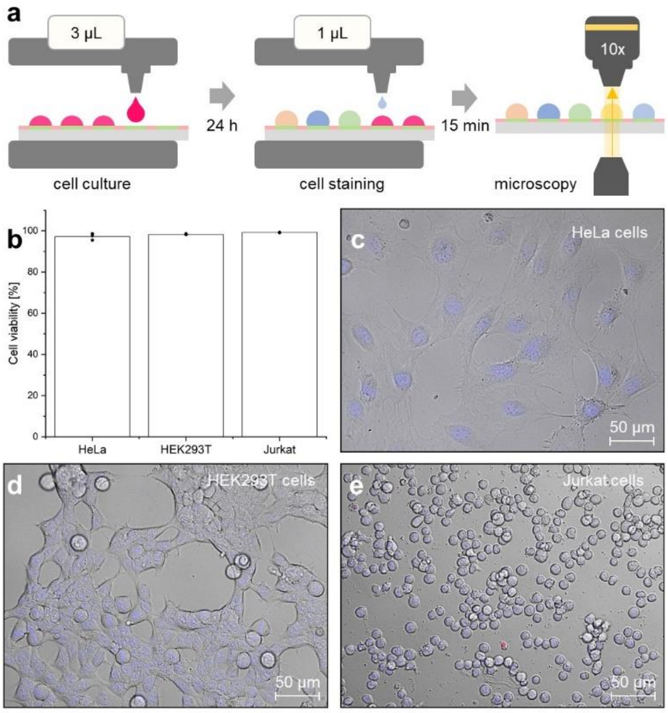

Supplementary Figure 11 | On-chip cell culture using dendrimer slides. (**a**) Schematic diagram showing the process of cell culture and viability tests. Cell suspensions were dispensed to each spot of a dendrimer-based chemBIOS slide and cultured at 37°C under 5% CO_2 for 24 h. Cell staining solution (Hoechst 33342 and propidium iodide) was dispensed to individual droplets and cell viability was evaluated by fluorescence microscopy. (**b**) Cell viability of on-chip cultured HeLa, HEK293T and Jurkat cells. (**c**) Microscopy images of stained (Hoechst 33342 and propidium iodide) HeLa, (**d**) HEK293T, and (**e**) Jurkat cells. Scale bar: 50 µm. Data represent mean ± standard deviation; each result based on triplicate control; n=3 independent experiments. Source data are provided as a Source Data file.

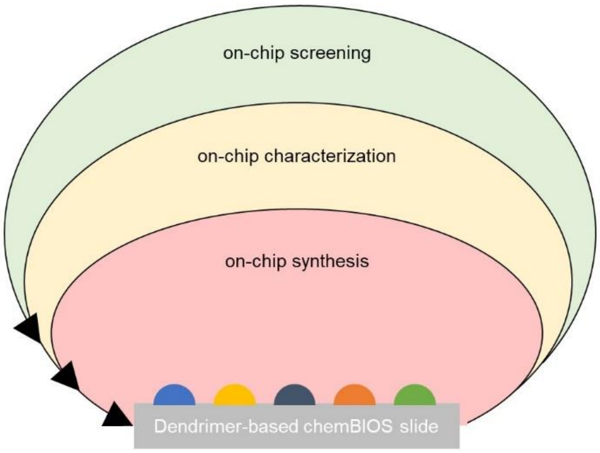

Supplementary Figure 12 | chemBIOS workflow. Schematic describing the chemBIOS workflow using a dendrimer-modified, omniphilic-omniphobic patterned slide that enables the handling of both low-surface tension liquids (organic solvents) and high-surface tension liquids (aqueous solutions / cell suspension) on the same substrate. On-chip synthesis, on-chip characterization and on-chip screening can be performed using one slide without the need of additional transfer steps.

Supplementary Tables

Supplementary Table 1 | Results of contact angle measurements. N/A: not tested. Each result based on triplicate control; n=3 independent experiments. Source data are provided as a Source Data file.

	solvent	$\varnothing\theta_{adv}$ [°]	$\sigma\theta_{adv}$ [°]	$\varnothing\theta_{stat}$ [°]	$\sigma\theta_{stat}$ [°]	$\varnothing\theta_{rec}$ [°]	$\sigma\theta_{rec}$ [°]
PFDT-modified G3 surface	H_2O	125	3	116	4	111	3
	DMSO	100	2	97	1	66	2
	DMF	103	1	91	4	67	3
	toluene	89	1	80	2	63	2
	n-hexadecane	86	1	81	2	74	1
	ethanol	80	1	72	2	40	0
Thioglycerol-modified G3 surface	H_2O	33	2	N/A	N/A	1	1
	DMSO	12	2	N/A	N/A	<1	<1
	DMF	4	1	N/A	N/A	<1	<1
	ethanol	3	1	N/A	N/A	<1	<1

Supplementary Table 2 | Results of surface roughness (Rq) measurements by AFM. Each result based on triplicate control; n=3 independent experiments. Source data are provided as a Source Data file.

Ø(Rq(unmod.)) [pm]	Ø(Rq(G0)) [pm]	Ø(Rq(G1)) [pm]	Ø(Rq(G2)) [pm]	Ø(Rq(G3)) [pm]
212±8	238±23	335±44	424±61	508±26
	**ΔRq(G0-unmod.) [pm]**	**ΔRq(G1-G0) [pm]**	**ΔRq(G2-G1) [pm]**	**ΔRq(G3-G2) [pm]**
	26	97	90	83

Supplementary Table 3 | Results of dendrimer layer thickness (d) measurements by AFM. Each result based on triplicate control; n=3 independent experiments. Source data are provided as a Source Data file.

Ød(G0) [nm]	Ød(G1) [nm]	Ød(G2) [nm]	Ød(G3) [nm]
1.27±0.22	2.03±0.05	2.99±0.19	4.19±0.49
	**Δd(G1-G0)**	**Δd(G2-G1)**	**Δd(G3-G2)**
	0.76	0.95	1.21

Supplementary Table 4 | Dendrimer layer thickness calculation.

	bond length [nm][1, 2]
C(sp3)-S	218
S-C(sp3)	218
C(sp3)-C(sp3)	154
C(sp3)-C(sp3)	154
C(sp3)-O	143
O-C(sp2)	143
C(sp2)-C(sp3)	150
C(sp3)-C(sp3)	154
C(sp3)-C(sp2)	150
C(sp2)-C(sp2)	134
Sum	1,618

Supplementary Table 5 | Mean MALDI-TOF MS Signal-to-Noise (S/N) ratios of lipidoid (L) 1, 2 and 3 measured on 2.83 mm spots before and after on-target washing. Each result based on triplicate control; n=3 independent experiments. Source data are provided as a Source Data file.

Amount on spot [fmol]	L1 before	L1 after	L2 before	L2 after	L3 before	L3 after
1,000	312±28	956±169	193±11	652±85	367±256	759±192
100	52±10	116±24	35±23	178±89	27±6	150±33
10	4±1	11±1	2±0	10±2	2±0	10±1
2	2±0	3±1		3±0		4±0

Supplementary Table 6 | Mean MALDI-TOF MS S/N ratios of lipidoid (L) 1, 2 and 3 measured on 900 µm and 500 µm spots after on-target washing. Each result based on triplicate control; n=3 independent experiments. Source data are provided as a Source Data file.

Amount on spot [fmol]	900 µm spots			500 µm spot		
	L1	L2	L3	L1	L2	L3
1,000	2,601±274	2,452±150	2,804±362	2,666±141	2,071±66	2,591±64
100	1,178±108	859±238	767±221	696±120	730±260	818±75
10	137±25	65±15	148±112	155±76	338±264	313±12
2	47±21	34±6	62±22	35±7	13±1	29±20
0.3	9±5	4±1	6±1	10±1	8±3	4±2
0.1	4±1	3±0	3±0	2±0	2±0	3±2
0.05	3±0	3±0		3±1	1±0	1±0

References

1. Fox, M.A. & Whitesell, J.K. Organische Chemie: Grundlagen, Mechanismen, Bioorganische Anwendungen. (Spektrum Akad. Verlag, 1995).
2. Lide, D.R. CRC Handbook of Chemistry and Physics: A Ready-Reference Book of Chemical and Physical Data. (CRC-Press, 1984).
