# Supplementary material for: A combined high-throughput and high-content platform for unified on-chip synthesis, characterization and biological screening
Source: Nat Commun. 2020 Oct 26;11:5391. doi: 10.1038/s41467-020-19040-0 (PMC7589500; doi:10.1038/s41467-020-19040-0)
Supplement: Supplementary file 3 — Reporting Summary [file 41467_2020_19040_MOESM3_ESM.pdf]

nature research

Corresponding author(s): Pavel A. Levkin

Last updated by author(s): Aug 18, 2020

Reporting Summary

Nature Research wishes to improve the reproducibility of the work that we publish. This form provides structure for consistency and transparency in reporting. For further information on Nature Research policies, see Authors & Referees and the Editorial Policy Checklist.

Statistics

For all statistical analyses, confirm that the following items are present in the figure legend, table legend, main text, or Methods section.

n/a	Confirmed	
☐	☒	The exact sample size (*n*) for each experimental group/condition, given as a discrete number and unit of measurement
☐	☒	A statement on whether measurements were taken from distinct samples or whether the same sample was measured repeatedly
☒	☐	The statistical test(s) used AND whether they are one- or two-sided<br>*Only common tests should be described solely by name; describe more complex techniques in the Methods section.*
☒	☐	A description of all covariates tested
☒	☐	A description of any assumptions or corrections, such as tests of normality and adjustment for multiple comparisons
☐	☒	A full description of the statistical parameters including central tendency (e.g. means) or other basic estimates (e.g. regression coefficient) AND variation (e.g. standard deviation) or associated estimates of uncertainty (e.g. confidence intervals)
☒	☐	For null hypothesis testing, the test statistic (e.g. *F*, *t*, *r*) with confidence intervals, effect sizes, degrees of freedom and *P* value noted<br>*Give P values as exact values whenever suitable.*
☒	☐	For Bayesian analysis, information on the choice of priors and Markov chain Monte Carlo settings
☐	☒	For hierarchical and complex designs, identification of the appropriate level for tests and full reporting of outcomes
☒	☐	Estimates of effect sizes (e.g. Cohen's *d*, Pearson's *r*), indicating how they were calculated

Our web collection on statistics for biologists contains articles on many of the points above.

Software and code

Policy information about availability of computer code

Data collection	Water Contact Angle: Krüss Advance 1.6.2.0; Krüss GmbH<br>AFM: MultiMode 8-HR; Bruker Corporation<br>ToF-SIMS: Surfacelab 7.0.106074; IONTOF GmbH<br>MS: flexControl 4.0.35; Bruker Corporation<br>IR: Opus 7.8; Bruker Corporation<br>UV-Vis: UV WinLab 6.0.4; PerkinElmer, Inc. / Gen5; BioTek Instruments, Inc.<br>Microscopy: BZ-II Viewer 1.5.0.0; Keyence Corporation
Data analysis	Water Contact Angle: Krüss Advance 1.6.2.0; Krüss GmbH<br>AFM: NanoScope Analysis 1.50 (build R3.119069); Bruker Corporation<br>ToF-SIMS: Surfacelab 7.0.106074; IONTOF GmbH<br>MS: flexAnalysis 4.0.14; Bruker Corporation<br>IR: Opus 7.8; Bruker Corporation<br>UV-Vis: Office 264 ProPlus Version 1908 Build 11929.20648; Microsoft / OriginPro 2019b Build 9.6.5.169; OriginLab Corporation<br>Microscopy: BZ-II Analyzer 2.1; Keyence Corporation / ImageJ 1.51s; Wayne Rasband National Institutes of Health / Office 264 ProPlus Version 1908 Build 11929.20648; Microsoft / OriginPro 2019b Build 9.6.5.169; OriginLab Corporation

For manuscripts utilizing custom algorithms or software that are central to the research but not yet described in published literature, software must be made available to editors/reviewers. We strongly encourage code deposition in a community repository (e.g. GitHub). See the Nature Research guidelines for submitting code & software for further information.

Data

Policy information about availability of data

All manuscripts must include a data availability statement. This statement should provide the following information, where applicable:

- Accession codes, unique identifiers, or web links for publicly available datasets
- A list of figures that have associated raw data
- A description of any restrictions on data availability

The data that support the findings of this study are available from the corresponding author upon reasonable request. The source data underlying Figs. 3a,b,e,g,i, 4b,c,f, 5b, and 6d and Supplementary Figs. 3, 5a–c, 6a–i, 7, 9b, and 11b and Supplementary Tables 1–4, 5 and 6 are provided as a Source Data file.

Field-specific reporting

Please select the one below that is the best fit for your research. If you are not sure, read the appropriate sections before making your selection.

☒ Life sciences ☐ Behavioural & social sciences ☐ Ecological, evolutionary & environmental sciences

For a reference copy of the document with all sections, see nature.com/documents/nr-reporting-summary-flat.pdf

Life sciences study design

All studies must disclose on these points even when the disclosure is negative.

Sample size	No sample size calculation was applied in this study to predetermine sample sizes for experiments using cell lines. A sample size of three was used as a starting point to evaulate the spread of the data (Casadevall A, Fang FC; Reproducible Science, Infect Immun, 2010 Dec; 78 (12):4972-4975). Experiments were repeated more often if necessary to provide results with statistical significance.
Data exclusions	No data was excluded from the analysis.
Replication	All attempts at replication were successful. The repeating numbers for each experiment are stated in the method part where it is applicable.
Randomization	No randomization was required as cell cultures were treated and grown identically apart from the experimental perturbation.
Blinding	No blinding was required as cell cultures were treated and grown identically apart from the experimental perturbation.

Reporting for specific materials, systems and methods

We require information from authors about some types of materials, experimental systems and methods used in many studies. Here, indicate whether each material, system or method listed is relevant to your study. If you are not sure if a list item applies to your research, read the appropriate section before selecting a response.

Materials & experimental systems

n/a	Involved in the study	
☒	☐	Antibodies
☐	☒	Eukaryotic cell lines
☒	☐	Palaeontology
☒	☐	Animals and other organisms
☒	☐	Human research participants
☒	☐	Clinical data

Methods

n/a	Involved in the study	
☒	☐	ChIP-seq
☒	☐	Flow cytometry
☒	☐	MRI-based neuroimaging

Eukaryotic cell lines

Policy information about cell lines

Cell line source(s)	HeLa (ATCC® CCL-2™); HEK293T (ATCC® CRL3216™); Jurkat (ATCC® TIB-152™)
Authentication	Non of the cell lines used were authenticated.
Mycoplasma contamination	All cell lines tested negative for mycoplasma contamination.
Commonly misidentified lines (See ICLAC register)	No misidentified cell lines were used.
